# Assessment of risky sexual behavior and practice among Aksum University students, Shire Campus, Shire Town, Tigray, Ethiopia, 2017

**DOI:** 10.1186/s13104-018-3199-7

**Published:** 2018-01-31

**Authors:** Awoke Kebede, Bogale Molla, Hadgu Gerensea

**Affiliations:** School of Nursing, College of Health Sciences and Referral Hospital, Aksum University, Aksum, Ethiopia

**Keywords:** Risk sexual behavior, Students, Ethiopia

## Abstract

**Objective:**

Having sex at early age, having multiple sexual partners, having sex while under the influence of alcohol or drugs and unprotected sexual behaviors are the common characteristics of risky sexual behavior which increases risk of individuals to sexuality and reproductive health problems. Risky sexual behavior is the most common problem in adolescents and young adults which may expose individuals for permanent social, economical, psychological and physical problem. So that this study focus on assessment of risk sexual behavior using institution based cross-sectional study design on 287 randomly selected subjects among Aksum University students.

**Results:**

Almost 60% students reported to have ever had sexual activity. Of which 86 (83.5%) and 112 (64.4%) reported having inconsistent condom use and multiple sexual partners respectively. Even though more than half of first sexual intercourse (61.5%) starts due to their desire but still peer pressure and alcohol have significant effect. Similarly the study indicated that a significant segment of students have risk sexual behaviors which increase individuals’ risk of acquiring HIV/AIDS. Unless appropriate age and institutional targeted interventions exist, certain behaviors can place the university students at greater risk of HIV infection and sexually transmitted disease.

## Introduction

Risk sexual behavior is commonly defined as behavior that increases susceptibility of an individual to problems related to sexuality and reproductive health. They include having sex at an early age, having multiple sexual partners, having sex while under the influence of alcohol or drugs and unprotected sexual behaviors [1, 2]. Current statistics on HIV/AIDS indicate that one-half of all new HIV infections worldwide occur among young people ages 15–24. Every minute, five young people worldwide become infected with HIV/AIDS [3].

The trends in sexual activity younger ages are increasing alarmingly in the world. In many countries the majority of young age people are sexually active before age of 20 and premarital sex is common among 15–19 years old [4]. World Health Organization reported in 2011 that unsafe sex was second among the top ten risk factors in the global burden of all diseases caused globally [5].

Students of higher learning institutions are people with multiple socio-cultural grounds begin autonomous life at younger age for first time and they rush to a range of maladaptive high-risk extracurricular activities like alcohol use, substance and sexual abuse [6].

Study done in America on adolescent and young students showed that both casual and chronic substance users are more likely to engage in high-risk behaviors such as unprotected sex [7].

Sub- Saharan Africa remains most severely affected with nearly one in every 20 adults 4.9% living with HIV and accounting for 69% of the people living with HIV worldwide [8]. Different studies also revealed that young adults in Sub Saharan Africa were also tend to engage in having multiple sexual partner, concurrent sexual partners and unprotected sexual intercourse [9, 10].

Adolescents represent a huge segment of potentially vulnerable population in Ethiopia and an increasing number of them are involved in unsafe sexual practices and hence face undesired health outcomes such as unplanned pregnancy, too early childbirth, unsafe abortion and sexually transmitted disease [11, 12]. There are evidences that show that some adolescents and young adults, who are left in the care of siblings without consistent adult supervision, have increased opportunities for sexual activity [13].

There are limited evidences on risk sexual behavior which is the focus this study. Therefore, the aim of this study is to assess the prevalence of risky sexual behavior among Aksum University students.

## Main text

### Study area

The study was conducted at Aksum University, Shire Campus which is one of the three campuses of Aksum University. It is located in northern Ethiopia, northwest zone of Tigray regional State, around 1078 kms from Addis Ababa, the capital city of Ethiopia and 60 km from the main campus, Aksum. The research was conducted from April 1 to May 1, 2017.

### Study design

Institutional based cross sectional study design was conducted.

### Sample size

A total of 313 samples were calculated using a single population proportion formula by assuming 5% marginal error and 95% confidence interval (σ = 0.05) and prevalence of the risk sexual behavior 31.4% [14] and by adding 10% of non-response rate.

### Sampling technique

Three departments were selected by lottery method. Then the allocated sample was proportionally distributed to the selected departments and year of studies based on number of students. Finally students were selected from each selected departments and year of study by simple random sampling method by taking list of students which belongs to selected departments of all years from the office of the registrar.

### Data collection instrument and techniques

A structured questionnaire was adopted and adapted from WHO sexual and reproductive health questioners and after review of relevant literatures and arranged according to particular objective it can address [15, 16]. Before actual data collection, the tool was pre-tested in Adigrat University. Two days training was given to all data collectors and supervisors prior to pretesting. Eight data collectors who had completed diploma in midwife were recruited. The data was collected through self-administer questionnaire.

### Data processing and analysis

Data was analyzed using SPSS by the principal investigators. Frequency and percentage of each variable is presented using tables and figures.

### Ethical consideration

Ethical approval was obtained from the college of the health science and referral hospital office of quality assurance enhancement. Permission to carry out this study was obtained from Aksum University of Shire Campus administration. Once permission was obtained from responsible body and written informed consent was asked from participants.

## Results

### Sociodemographic characteristics

Majority the respondents were males in sex and orthodox follower in religion. Majority of the respondents’ age ranges from 21 to 23 years. More than half of the respondents are from rural. Concerning ethnicity, majority were Tigray followed by, Amhara. With regard to monthly income, majority earns greater than 300 ETB (Table 1).

**Table 1:** Shows sociodemographic characteristic of respondents among Aksum University, Shire Campus regular students, Shire Town, May, 2017 (n = 287)

S. no	Variables	Categories	Frequency (n = 287)	Percentage
1	Sex	Male	171	59.6
		Female	116	40.4
2	Age	18–20	40	13.9
		21–23	156	54.9
		24–26	75	26.4
		≥ 27	16	5.6
3	Previous place of residence	Urban	137	47.7
		Rural	150	52.2
4	Religion	Orthodox	203	71.0
		Muslim	46	16.0
		Protestant	32	11.0
		Others	6	2.0
5	Monthly income of the respondents	≤ 300 (ETB)	126	43.9
		> 300 (ETB)	161	56.1
5	Occupation of mother	Housewife	174	60.6
		Employed	33	11.5
		Merchant	24	8.4
		Farmer	52	18.1
		No mother	4	1.4
6	Parents income per month	< 2000 ETB	44	15.3
		2000–3000 ETB	89	31.0
		> 3000	154	53.7
7	Ethnicity	Tigray	124	43.0
		Amara	98	34.0
		Oromia	34	12.0
		Somali	17	6.0
		Others	14	5.0

### Substance use

The Substance use by respondents was assessed with respect to drinking alcohol, chewing chat and smoking cigarette. This study finding showed that from the total respondents about 46.7, 14.3, and 16.8% of the respondents reported they had experienced drinking alcohol, chewing chat, and smoking cigarette respectively (Table 2).

**Table 2:** Sexual history, contraceptive, substance and condom use among respondents of Aksum University Shire Campus, undergraduate students, Northern Ethiopia, May, 2017, (n = 287)

Variables	Categories	Frequency	Percent
Ever had sexual intercourse	Yes	174	60.6
	No	113	39.4
With whom first sex was made	Boy or girl friend	121	69.5
	Stranger	13	7.5
	Relatives	11	6.3
	Sex worker	12	6.9
	Don’t know	17	9.8
Type of sex made	Vaginal	174	100.0
	Anal	11	6.3
	Oral	9	5.2
Age at first sex	< 18	66	37.9
	> 18	89	51.2
	Don’t remember	19	10.9
Number of sexual partners over life time	One	62	35.6
	More than one	112	64.4
Accept premarital sex practice	Yes	129	45.0
	No	158	55.0
Condom use	Yes	103	59.2
	No	71	40.8
Frequency of condom use	Always	47	16.5
	Most of the time	92	32.0
	Some times	148	51.5
Contraceptive usage among started sex	Used	95	54.6
	Not used	79	45.0
Drinking alcohol	Yes	134	46.7
	No	153	53.3
Smoking cigarette	Yes	48	16.7
	No	239	83.3
Chewing chat	Yes	41	14.2
	No	246	85.0

### Sexual practice

Out of the total respondents, 174 (60.6%) of students had sexual experience. From the total respondents who had practiced sexual intercourse 66 (37.9%) of them reported that their first sexual intercourse was before age of 18 years while 89 (51.2%) of them had their first sex at above or equal to 18 years. All of the respondents with sexual experience 174 (100%) has practiced vaginal sex, followed by anal and oral in which 6.3 and 5.2% respectively (Table 2).

### Premarital sex and contraceptive use

From the study participants nearly half (45.0%) accept (agreed) with premarital sexual practice. Among those who had sexual intercourse more than half 95 (54.6%) reported they had used one of the contraceptive methods at the time of their first sexual intercourse (Table 2).

### Condom use

From sexually active 174 students, 103 (59.2%) had used condom at least once in their life time during sexual intercourse. Among those above three quarter (83.5%) were identified as using condom inconsistently (Table 2).

### Reason for initiation of first sex

Respondents were asked about the main reasons for initiation of sex, and majority of the respondents were based on personal desire 107 (61.5%). They initiate sexual intercourse based on their willingness like, fail in love, promising word from partner for marriage, curiosity or to see what it is, whereas 67 (38.5%) initiated without their willing like, peer pressure 37 (21.3%), influence of alcohol 17 (9.6), economic problem 8 (4.6%), influence of chat or drug 3 (1.7%), and others 2 (1.2%) (Table 3).

**Table 3:** Reasons for initiation of first sex in undergraduate students of Aksum University Shire Campus, Northern Ethiopia, May, 2017, (n = 287)

Variables	Categories	Frequency	Percent
The main reason to start sexual intercourse at first time	Desire to sexual experiment	107	61.5
	Peer-pressure	37	21.3
	Influence of alcohol	17	9.6
	Influence of chat or drug	3	1.7
	Economic problem	8	4.6
	Don’t know	2	1.2

## Discussion

Even though assessment of risky sexual behavior is somewhat challenging, particularly when adolescents and young adults are involved, this is the only available window to assess the risk sexual behavior in the study area. Similarly young adults and adolescents are the main risky group and are higher proportion, this finding gives clue for policy makers and higher officials to take some measures on the specific finding.

This study revealed that 60.6% of respondents ever had sexual activity. This finding is higher than the findings from Jimma and Haramaya University, in which (26.9%) and (33.5%) of students ever had sexual intercourse, respectively [17, 18]. This might be partly due to difference in the study time and there is also slight difference in age.

In this study, about 37.9% of the respondents had experienced premarital sex before the age of 18 years. This finding is lower than the finding from study conducted among male college students of Kathmandu Nepal, showed that about 63.7% students were had sex before the age of 18 [19]. This might be due to the difference in awareness and cultural value of the respondents towards disadvantage of premarital sex and most sexual practice in Ethiopia is hidden or private domain. More over there is also difference in age distribution of the study subjects.

This study also found that Condom utilization at the time of intercourse among who had sexual intercourse was about 59.2%, which is consistent with a study done in Jimma University which revealed that 69.1% utilized condom [1]. This might be due the similarity in accessibility of condom freely. This is also comparable with a study done in Ugandan universities which revealed that 50.0% utilized condom [20]. This may be due to the countries economical status and geographical location.

However, this study showed that the prevalence of inconsistent condom use was 83.5% despite the expectation of prevention awareness among university students; the prevalence is higher as compared to a study done in United States of America which shows that 64.0% of participants use condom inconsistently [21]. This might be due the magnitude of causal sexual practice and the difference in attitude of the respondents for different peoples, which indicates inadequacy of knowledge about reproductive health risks including HIV/AIDS, STI, unwanted pregnancy and its complications which have grave consequences on the students themselves and country as whole.

In this study 64.4% of study participants have more than one sexual partner which is more than twice than a study done in Haramaya and Jimma Universities, in which 11.5 and 28.3% had multiple sexual partners respectively [17, 18]. When we compare it from abroad, it is above twice from the study done in Uganda in which 24.0% of the participants have more than one sexual partner [20]. This difference may be due to lack of awareness on the disadvantage of having multiple sexual partners and lack of youth reproductive health services. Similarly the study difference may be related with period of study.

Moreover, the study revealed that nearly half about 46.7% of the participants uses alcohol which is comparable with a study done in Haramaya University in which 41.7% uses alcohol, but the prevalence of chat chewing is lower than by half (14.3%) from the study conducted in Haramaya University in which 30.3% of study participants uses chat [17]. This may be due to the cultural background of the study area and the accessibility of the substance respectively. Since chat is accessible in Harramaya but not in Aksum university.

In this study from the total of those participants with sexual activity the reason to start sexual intercourse was personal desire (61.5%) which is higher than the study done in Zambia in which 48.0% of the participants were due to personal desire. Secondly, peer pressure was the second most important factor to start sex (21.3%) which is comparable with the study done in Zambia in which 18.0% of the participants were practicing due to peer pressure [22]. This result may be an indicator for the influence of peer pressure and the need of awareness creation on friendship.

In this study 6.9% of the participants have their first sex with commercial sex worker which is lower than the study done in Haramaya University in which 16.3% of the participants have their first sex with commercial sex workers [17]. This may be due to limited number of bars, coffee houses and commercial sex workers.

For many times, interventions in the universities have focused on abstinence as policy. But as we have seen in this study 60.6% have already been in sexual activity in which abstinence by itself does not work. Again 64.4% ever had a practice of sex with multiple sexual partners and 83.5% use condom inconsistently. This shows as limiting sexual practices and not availing condom and other services do not hamper the students from performing sexual intercourse anywhere else unless their behavior is modified.

## Conclusion and recommendations

Significant segment of students have risk sexual behaviors which increase individuals’ risk of acquiring HIV/AIDS. Substance use and peer pressure were revealed as predisposing factors for the existence of sexual risk behaviors. Unless appropriate age and institutional targeted interventions exist, certain behaviors can place the university students at greater risk of HIV infection and Sexually transmitted disease. So, universities should target to ensure healthy behavioral modification with availing necessary services like condom and contraceptive.

## Limitation

There is a possibility of both under reporting of risk sexual behavior, because study the topic by itself assesses personal and sensitive issues related to sexuality.

## Abbreviations

HIV: human immunodeficiency virus
AIDS: acquired immunodeficiency virus
STI: sexually transmitted infection
STDs: sexually transmitted diseases
WHO: World Health Organization
ETB: Ethiopian Birr
